# Graded levels of phytase on performance, bone mineralization and carcass traits of broiler fed reduced dicalcium phosphate

**DOI:** 10.5713/ajas.18.0228

**Published:** 2018-07-26

**Authors:** Henrique Barbosa de Freitas, Karina Márcia Ribeiro de Souza Nascimento, Charles Kiefer, Gilson Alexandre Gomes, Tiago Tedeschi dos Santos, Elis Regina Moraes Garcia, Thiago Rodrigues da Silva, Luanna Lopes Paiva, Patrícia Rodrigues Berno

**Affiliations:** 1 Faculty of Veterinary Medicine and Animal Sciences/Federal University of Mato Grosso do Sul, Campo Grande 79002, Brazil; 2 ABVista, Marlborough SN8 4AN, UK; 3 Graduate Program in Animal Science, Mato Grosso do Sul State University, Aquidauana 79200, Brazil

**Keywords:** Amino Acids, Enzyme, Phosphorus, Phytic Acid

## Abstract

**Objective:**

This study was conducted to assess increasing doses of phytase added to broiler diets formulated with different levels of available phosphorus (avP), calcium (Ca), and sodium (Na), and the respective effects on performance parameters, quantitative carcass characteristics, ash and phosphorus deposition in tibia and weight of organs.

**Methods:**

Three different matrices were assumed for phytase with the following nutritional values: matrix A (MT A): 0.165% Ca, 0.150% avP, and 0.035% Na; matrix B (MT B): 0.215% Ca, 0.195% avP, and 0.045% Na; matrix C (MT C): 0.245% Ca, 0.225% avP, and 0.053% Na. There were six different diets: No phytase (formulated to meet the nutritional requirements); phytase 500 FTU/kg+MT A; phytase 1,000 FTU/kg+MT A; phytase 1,500 FTU/kg+MT A; phytase 1,000 FTU/kg+MT B and phytase 1,500 FTU/kg+MT C.

**Results:**

There was no significant phytase influence on performance, quantitative carcass characteristics, ash and phosphorus deposition in tibia and weight of the organ throughout the study period, however, it was possible to observe a tendency of improvement in body weight corrected feed conversion for broilers fed the phytase 1,500+MT C diet, where potentially these birds were more efficient on utilize phytic phosphorus and other nutrients bounded to phytate molecule, translating into improvement in performance, and there was also a non significant numerical improvement in body weight corrected feed conversion of broilers fed this diet.

**Conclusion:**

Broilers fed with diets formulated with different levels of avP, Ca, and Na and increasing doses of phytase have shown no change on performance, quantitative carcass characteristics, ash and phosphorus deposition in tibia and weight of organs.

## INTRODUCTION

Phosphorus is the second most abundant element in animal tissues, with 80% of the total phosphorus deposited in the skeletal system and the remaining distributed in fluids and other tissues [1]. In addition to structural functions in the body, phosphorus is involved in several metabolic processes, such as the development of skeleton, muscle tissue and eggs; it is an element of nucleic acids; it is essential in the control of cell metabolism; assists osmotic maintaining, the acid-base balance, the synthesis of membrane phospholipids; participates in energy transfer as a component of the adenosine triphosphate molecule and activates many enzymatic processes [2,3].

Nowadays, it is common to use phosphate to meet the phosphorus requirement, however, this source is not easily renewable, making it a high cost ingredient for monogastric diets [4]. Besides its high cost of inclusion, the negative effects overcome the productive performance fall, where high concentrations of inorganic phosphorus in excreta can cause issues to the environment, such as eutrophication and nitrification, decreasing the amount of oxygen dissolved in waters of rivers and lakes, and leading, even, to the death of fish and aquatic organisms, and possibly to the soil contamination by this component [5].

Broiler diets are basically formulated with high quantities of corn and soybean meal, however, about two-thirds of phosphorus are unavailable, as the phytic acid molecule or phytate plays a role of phosphorus reserve in the plant. Considering that birds are unable to hydrolyze efficiently this molecule present in the grain, as they do not produce sufficient amount of endogenous phytase, the utilization and the digestibility of phytic phosphorus and bounded nutrients are affected [6]. Not only phosphorus, but also calcium (Ca), magnesium, potassium, cobalt, manganese and zinc are minerals that may also be bounded [7]. Besides the development of stable complexes with minerals, phytic acid has a high capacity of binding amino acids to its molecule, so its presence in the diet can decrease the digestibility of minerals, lipids, starch and proteins, by reducing the digestive enzymes efficiency, such as lipase, α-amylase and pepsin [8], translating into losses in broiler growth.

Considering that phytic acid is an antinutritional factor and the cost of inorganic phosphorus to supplement diets is high, the search for viable solutions to improve the utilization of phytic phosphorus and nutrients by the birds became constant. Positive results have been reported that confirm the beneficial effects of phytase on the hydrolysis of phytic acid molecule, improving the digestibility of minerals, amino acids and energy, translating into higher nutrient utilization by broilers, and positively influencing bone ash concentration as well as increasing bone breaking strength [9–13].

More recently poultry producers are benefiting from phytase superdosing, which is considered to provide extra-phosphoric effects. This new concept is characterized by using the enzyme in addition to its conventional use in industry (500 FTU/kg), aiming a more efficient hydrolysis of phytic acid, with a decrease of its antinutritional effects. Consequently, broilers show higher performance, linked to the maximum utilization of minerals and nutrients [13,14]. High doses, beyond improving the birds’ growth, produce economic advantages, as more robust nutritional matrix can be taken into account (available phosphorus [avP], Ca, and Na), where the partial or total replacement of dicalcium phosphate in diet may be an alternative, translating into improved economic efficiency [6]. On this way, the aim of this study was to test graded levels of phytase, either applied on diets gradually decreased on avP, Ca, and Na, or on top of diets containing 500 FTU/kg of phytase, and the respective effects on performance, tibia ash and phosphorus, weight of organs and carcass yields of broilers from 1 to 42 days of age.

## MATERIALS AND METHODS

### Animals, diets and experimental design

All the procedures adopted in the present study were approved by Ethics Committee of Federal University of Mato Grosso do Sul for animal use.

A total of 900 male day-old Cobb 500 chicks, (weighing 45 g on average at placement) were allocated according to a completely randomized design, in six treatments with five replications of 30 birds each.

Experimental diets were formulated to meet the nutritional requirements of broilers, as determined by Rostagno et al [15], except for Ca, avP, and Na, and the dietary levels of minerals were reduced according to the nutritional matrix recommended by the enzyme manufacturer to 500, 1,000, and 1,500 FTU/kg of phytase (Table 1).

The trial period was divided into three phases: starter (1 to 21 days), grower (22 to 33 days) and finisher (34 to 42 days). Diets were corn/soybean meal based (Tables 2, 3, 4), and kaolin was used as inert in the formulations, allowing constant inclusion of most ingredients, varying only the inclusions of dicalcium phosphate, limestone, salt and inert.

Phytase added to the experimental diets was from *Escherichia coli* (*E. coli*) produced in *Thricoderma reesei* (Quantum Blue, AB Vista, Marlborough, UK), replacing part of the inert ingredient in the diets. Prior to trial commencement the commercial enzyme preparation was analyzed, and then the dosage in the experimental diets was adjusted according to the analysis of pure product. Along with phytase, xylanase (Econase XT, AB Vista, Marlborough, UK) at 16,000 BXU/kg was incorporated to all experimental diets, and a contribution of 75 kcal/kg of metabolizable energy from this enzyme was considered. Diets were thereafter analyzed for phytase and xylanase activities.

### Experimental procedures and conditions

Pens were equipped with two 100 W incandescent bulbs for chicks heating, a tubular feeder, a bell drinker and the litter material was pine shavings. In addition, gas heaters were distributed inside the shed to serve as a supplementary environmental heating system during the starter phase.

The adopted lighting program was continuous (24 L:0 D) across the experimental period. Temperature and humidity were monitored at 7 am and 5 pm, daily, through a digital thermometer of minimum and maximum, installed inside the pen, at bird’s height.

Feed intake was measured through the difference between the allowance and leftover for each feeding phase, and at the beginning of the trial and the end of each feeding phase pen body weight determined. Mortality was measured daily. Average body weight gain, average feed intake, was then calculated for the periods (1 to 21; 1 to 33; and 1 to 42 days). Mortality corrected feed conversion was calculated according to Sakomura and Rostagno [16]. Additionally, body weight corrected feed conversion was determined according to Patricio et al [17].

At 21 days of age, one bid per replication with body weight close to the average of the experimental unit (±10%), was selected, submitted to fasting for 8 hours, euthanized by cervical dislocation followed by bleeding and evisceration. After that, left tibias were collected, defatted and analyzed for ash and phosphorus, according to Silva and Queiroz [18]. Organs were weighed to determine relative weight of organs (proventriculus, gizzard, small intestine, pancreas, heart, and liver). The relative organ weights were expressed as the absolute weight of each organ divided by chicken weight before slaughtering and the result was multiplied by 100.

At 42 days of age, two birds per replication with body weight close to the average of the experimental unit (±10%) were selected, euthanized by cervical dislocation followed by bleeding, scalding, plucking and evisceration. The fat close to cloaca and gizzard (abdominal fat) was removed and weighed. Feetless, headless, neckless carcasses were weighed and cut in breast, thigh+drumstick, wings and back. Carcass, feet and head+neck yields were determined by the ratio between absolute weight of the carcass and its cuts and body weight before slaughtering. Breast, thigh+drumstick, wings, back, head+neck, and abdominal fat yields were determined in relation to the eviscerated carcass (feetless, headless, neckless) weight.

### Statistical analyses

The performance parameters, ash and phosphorus deposition in tibia, weight of organs and carcass yields were submitted to analysis of variance by the general linear model procedure of the SAS program, with the pens being considered as the experiment unit. Trends were discussed considering p<0.10. In case of significant differences (p<0.05), the means of the treatments were compared by the Tukey’s test at 5% probability.

## RESULTS

### Growth performance

Feed intake, body weight, body weight gain, feed conversion and livability results of all diets supplemented with phytase were like the diet without phytase, in all feeding phases (Table 5).

The best nutrient utilization in the diet with phytase addition was evident especially in the last two phases, in which the phytase 1,500+MT C (reduction of 0.225% avP; 0.245% Ca; and 0.053% Na, with 1,500 FTU/kg phytase) did not decreased broiler performance, enabling partial and total replacement of dicalcium phosphate on starter, grower and finisher phases, respectively.

Body weight corrected feed conversion tended to improve (p = 0.08) with the phytase 1,500+MT C (decrease of 0.225% avP; 0.245% Ca; and 0.053%, with phytase at 1,500 FTU/kg) diet, although significant differences between weight gain and feed conversion were not observed.

### Tibia ash and phosphorus, weight of organs and carcass yields

The analysis of ash and phosphorus content of tibias at 21 days of age were not influenced by the levels of phytase (Table 6).

No effects of treatments were observed on the relative organ weights of broilers at 21 and 42 days (Table 7), except for the relative weight of the liver at 42 days (p<0.05). The phytase 1,000+MT B diet produced higher relative liver weights in relation to the diet without phytase.

Characteristics of carcass and cuts were not influenced by the addition of phytase in the diets (Table 8).

## DISCUSSION

Although there were no statistical differences between the performance variables, the decreasing mineral levels in the diets (avP, Ca, and Na) associated with phytase addition resulted in adequate broiler growth. Probably the results are associated with the phytase supplementation, which promoted hydrolysis of phytic acid-phosphorus compound, releasing phosphorus and other nutrients bounded, resulting in maintenance of performance, without decreasing the weight gain of broilers fed a diet with reduced nutritional levels of minerals.

The inclusion of phytase in the diet promoted maintenance of broiler performance due to the efficient hydrolysis of phytate and therefore phosphorus, Ca, Na, zinc, iron and magnesium were available. In addition to the minerals, possibly the amino acids like glycine, serine, threonine and proline were also available for broilers [6,13].

The dicalcium phosphate used in broiler diets is a non-renewable resource, which makes it difficult to be obtained, and consequently, presents high cost to purchase. Based upon that, we note that the partial or total replacement of dicalcium phosphate in diets with added phytase can save broiler feed costs, besides reducing environmental pollutant capacity of broiler excreta, as it decreases the use of inorganic phosphorus source [5].

Similar effects found in this study were observed when broilers were fed diets containing phytase, confirming the enzyme’s ability in hydrolysis partially or totally the molecule of phytic acid, to maintain the performance without losses in productive parameters [19,20].

However, improved performance of broilers at 21 days of age fed diets supplemented with phytase was reported by Fukuyama et al [21], where they observed an increase of 15% in weight gain and an improvement of 8% in feed conversion, when broilers were fed initial diets supplemented with 1,000 FTU/kg compared to the diet without phytase supplementation. However, the diets from this study did not consider lower nutritional mineral levels under the same intensities as the present study.

Similarly, Bradbury et al [14] have noted positive effects on the performance parameters when the initial diets were supplemented with 1,000 FTU/kg of phytase, indicating an increase in nutritional digestibility and, consequently, in broiler growth at 28 days of age. However, only the nutritional matrix of calcium was considered in that study (30% reduction), while, in the present search, the nutritional matrix was adjusted to upper levels, beyond including other minerals (avP, Ca, and Na), to take advantage of its maximum enzymatic potential and as a possible replacement for the inorganic source of minerals.

On the other hand, Karadas et al [10] using phytase from *E. coli* at 12,500 FTU/kg, associated with avP nutritional matrix of 0.20%, observed an improvement in weight gain and feed conversion when compared to broilers fed diets without phytase or with 500 and 1,000 FTU/kg. The same authors state that these results are due to the fact that young birds can respond positively to enzymatic activities higher than 1,000 FTU/kg, consequently high levels of phytase can cause beneficial effects on performance and nutrient digestibility when compared to lower levels.

Broilers at 42 days of age fed with the phytase 1,500+MT C diet were able to maintain adequate performance, even with the robust phytase nutritional matrix. It’s likely that these birds were more efficient on utilizing phytate-P and other nutrients bounded to phytate molecule [22].

Nevertheless, better performance of broilers at 42 days was observed when they were fed with diets supplemented with phytase. Liu et al [23] noted that phytase supplementation at 1,000 FTU/kg increased phytic phosphorus in the diet and improved weight gain and feed conversion when compared to broilers not fed phytase.

Similarly, Campasino et al [24] evaluated the supplementation of 400, 800, 1,200, and 1,600 FTU/kg of phytase in broiler diets, with the nutritional matrix of 0.14% Ca; 0.13% P; and 0.03% Na, at 42 days of age and reported linear improvement in feed conversion of broilers supplemented with increasing levels of the enzyme.

The results of the present trial indicate improvement in the efficiency of dietary phytic phosphorus utilization through phytase supplementation, as the percentage of phosphorus in the tibia of broilers fed with phytase 1,500+MT C diet was statistically similar to the birds fed diet without phytase (8.26% ×8.50%). These results confirm the phytase ability to release mainly phosphorus and calcium phytic for adequate bone mineralization, even considering the high nutritional challenge imposed to birds fed with the matrix C diet (diets reduced by 0.225% avP; 0.245% Ca and 0.053% Na, and supplemented with 1,500 FTU/kg of phytase), which caused no reduction on bone mineralization, showing the ability of the enzyme to release minerals thereby, fulfilling the requirements for such nutrients.

The percentage of ash in the tibia as a good indicator of bone mineralization in broilers and a very sensitive parameter to determine the effect of adding phytase on mineral availability in the diet [25]. The results suggest that supplementation of 1,500 FTU/kg in broiler diets lowering by 0.225% avP, 0.245% Ca; and 0.053% Na was effective in hydrolysis of phytic acid and release of phosphorus and the minerals considered. The results indicate the possibility of partial replacement of inorganic phosphorus sources by the enzyme, in the starter and grower phases, and total replacement in the finisher phase, as the replacement of dicalcium phosphate by 1,500 FTU/kg of phytase has not affected the bone mineralization of broiler tibia.

Evaluating the effect of 500, 700, and 1,000 FTU/kg of phytase added to broiler diets, Fukuyama et al [21] confirmed the results found in this study, where ash deposition was not influenced by the enzyme. Similarly, Onyango et al [26] analyzed the inclusion of 1,000 FTU/kg of phytase in broiler diets considering a nutritional matrix of 0.38% total phosphorus and 0.50% calcium and observed similar concentrations of tibia ash at 21 days of age when compared to diet without phytase.

However, other studies reported negative effects of phytase addition on phosphorus deposition in tibia. Fukuyama et al [21] evaluating the deposition of phosphorus in tibia of broilers at 21 days, found that the addition of 1,000 FTU/kg of phytase negatively influenced the concentration of this mineral, and, at this level the amount deposited in the bone was significantly less than that from birds fed diet without phytase added. Laurentiz et al [27] observed decrease in deposition of phosphorus in tibia, when birds were fed with diets containing phytase and reduced levels of avP. The results presented by these authors show that the addition of phytase in the diets was not reflected in better deposition of phosphorus in tibia, likely because of the utilization of this mineral in other vital body processes, such as growth and deposition of muscle tissue.

The phytase 1,000+MT B diet presented higher relative liver weight in relation to the diet without phytase. This may have happened because these birds were slightly lighter than birds of other treatments. Other results obtained show similar development of examined organs, confirming the efficacy of phytase on utilization of dietary nutrients, avoiding then, the depressor effect of phytic acid molecule on the body and organs development.

Confirming the results found in this study, Nunes et al [28] evaluated the phytase complex utilization on the development of broiler organs, and there was no influence of enzyme addition on the growth of organs and intestinal biometrics.

Characteristics of carcass and cuts were not influenced by the addition of phytase, however the addition of phytase reduced the antinutritive effects of phytate on the weight of cuts. Besides that, the inclusion of phytase increased positively the ileal digestibility of various amino acids, such as lysine, arginine and histidine [12].

These results are according to the observations of Gomide et al [29], where the decrease of calcium and avP associated with phytase supplementation has not provided improvements in carcass and chicken breast yield of broilers fed diets containing phytase at 500 FTU/kg.

Evaluating the effect of phytase at 400, 800, 1,200, and 1,600 FTU/kg in diets with lower levels of avP and calcium, Campasino et al [24] did not report an influence of phytase levels on carcass and breast chicken yield of broilers at 42 days of age fed diets containing enzyme. The lack of phytase effect on the carcass and cuts characteristics evaluated in this study were likely justified by the similar performance presented by birds between the supplementation levels associated with the nutritional matrix.

## CONCLUSION

In summary, the results of this trial suggest that broilers fed diets supplemented with increasing doses of phytase and containing different levels of avP, Ca, and Na present no change in performance. However, phytase added to the diet at 1,500 FTU/kg shows to be effective, making it possible to consider more robust phytase nutritional matrices, since it provides similar results to birds fed nutritionally adequate diets and without phytase supplementation. Thus, it becomes possible to replace, partially or completely, dicalcium phosphate and may therefore reduce feed costs in broiler production, without decrease performance parameters and it allows the reduction of nutrients excreted by the birds to the environment as well.

## Figures and Tables

**Table 1:** Experimental diets according to phytase nutritional matrix and reduction of nutrients

Experimental diets	Phytase nutritional matrix1)	Dietary nutrient down specification2) (%)			Phytase dose (FTU/kg)

		Available phosphorus (avP)	Calcium (Ca)	Sodium (Na)	
1	-	-	-	-	-
2	A	0.150	0.165	0.035	500
3	A	0.150	0.165	0.035	1,000 (500 on top)
4	A	0.150	0.165	0.035	1,500 (1,000 on top)
5	B	0.195	0.215	0.045	1,000
6	C	0.225	0.245	0.053	1,500

1) Nutrient down specification in relation to control diet (1) in experimental diet 1 there was no reduction of avP, Ca, and Na.

2) Reduction of dietary avP, Ca, and Na levels followed the supplier recommendation for (A) 500; (B) 1,000; and (C) 1,500 FTU/kg of phytase.

**Table 2:** Nutritional composition and calculated values of broiler experimental diets (1 to 21 days)

Items	Experimental diets1)					

	No phytase	Phytase 500 +MT A	Phytase 1,000 +MT A	Phytase 1,500 +MT A	Phytase 1,000 +MT B	Phytase 1,500 +MT C
Ingredients (%)						
Corn 7.88%	57.31	57.31	57.31	57.31	57.31	57.31
Soybean meal 46%	35.12	35.12	35.12	35.12	35.12	35.12
Soybean oil	2.47	2.47	2.47	2.47	2.47	2.47
Dicalcium phosphate	1.56	0.75	0.75	0.75	0.51	0.35
Kaolin	1.00	1.80	1.79	1.78	2.04	2.18
Limestone	0.95	1.03	1.03	1.03	1.06	1.09
Initial Vit+Min supplement2)	0.40	0.40	0.40	0.40	0.40	0.40
Salt	0.38	0.30	0.30	0.30	0.27	0.25
DL-methionine	0.32	0.32	0.32	0.32	0.32	0.32
L-lysine HCL	0.25	0.25	0.25	0.25	0.25	0.25
Sodium bicarbonate	0.15	0.15	0.15	0.15	0.15	0.15
L-threonine	0.08	0.08	0.08	0.08	0.08	0.08
Xylanase	0.01	0.01	0.01	0.01	0.01	0.01
Phytase	0.00	0.0075	0.0150	0.0225	0.0150	0.0225
Total	100.00	100.00	100.00	100.00	100.00	100.00
Calculated values						
Metabolizable energy (Kcal/kg)3)	2,975	2,975	2,975	2,975	2,975	2,975
Crude protein (%)	21.20	21.20	21.20	21.20	21.20	21.20
Available phosphorus (%)	0.401	0.251	0.251	0.251	0.206	0.176
Calcium (%)	0.841	0.676	0.676	0.676	0.626	0.596
Sodium (%)	0.210	0.175	0.175	0.175	0.165	0.158
Chlorine (%)	0.279	0.226	0.226	0.226	0.211	0.201
Methionine+cysteine (%)4)	0.876	0.876	0.876	0.876	0.876	0.876
Methionine (%)4)	0.760	0.760	0.760	0.760	0.760	0.760
Lysine (%)4)	1.217	1.217	1.217	1.217	1.217	1.217
Threonine (%)4)	0.791	0.791	0.791	0.791	0.791	0.791
Tryptophan (%)4)	0.232	0.232	0.232	0.232	0.232	0.232
Dietary electrolyte balance (mEq/kg)	219.62	219.22	219.22	219.22	219.11	219.02
Phytase (FTU/kg)5)	0	500	1,000	1,500	1,000	1,500
Xylanase (BXU/kg)6)	16,000	16,000	16,000	16,000	16,000	16,000
Analyzed values						
Phytic phosphorus (%)	0.220	0.240	0.240	0.230	0.240	0.260
Phytase (FTU/kg)	0	515	1,128	1,370	1,043	1,345
Xylanase (BXU/kg)	15,850	17,050	18,950	18,350	16,150	17,200

1) Matrix A (MT A): 0.165% Ca, 0.150% avP, and 0.035% Na; matrix B (MT B): 0.215% Ca, 0.195% avP, and 0.045% Na; matrix C (MT C): 0.245% Ca, 0.225% avP, and 0.053% Na.

2) Initial Vit+Min supplement – levels per kg of feed: 450.75 g methionine; 65.25 g choline; 2,750,000 UI vitamin A; 500,000 UI vitamin D_3_; 4,000 UI vitamin E; 375 mg vitamin K_3_; 300 mg vitamin B_1_; 1,125 mg vitamin B_2_; 500 mg vitamin B_6_; 4,000 mcg vitamin B_12_; 8,750 mg niacin; 2,300 mg pantothenic acid; 100 mg folic acid; 15 mg biotin; 7,500 mg iron; 2,250 mg cupper; 15 g manganese; 15 g zinc; 250 mg iodine; 62.5 mg selenium; 2,500 mg avilamycin; 10 g nicarbazin; 3,750 mg semduramicin.

3) Nitrogen-corrected apparent metabolizable energy.

4) Digestible amino acid.

5) Garantee levels per g of phytase: 5,000 FTU.

6) Garantee levels per g of xylanase: 16,000 BXU.

**Table 3:** Nutritional composition and calculated values of broiler experimental diets (22 to 33 days)

Items	Experimental diets1)					

	No phytase	Phytase 500 +MT A	Phytase 1,000 +MT A	Phytase 1,500 +MT A	Phytase 1,000 +MT B	Phytase 1,500 +MT C
Ingredients (%)						
Corn 7,88%	60.37	60.37	60.37	60.37	60.37	60.37
Soybean meal 46%	31.64	31.64	31.64	31.64	31.64	31.64
Soybean oil	3.34	3.34	3.34	3.34	3.34	3.34
Dicalcium phosphate	1.34	0.53	0.53	0.53	0.29	0.13
Kaolin	1.00	1.81	1.80	1.79	2.04	2.18
Limestone	0.89	0.98	0.98	0.98	1.00	1.03
Initial Vit+Min supplement2)	0.30	0.30	0.30	0.30	0.30	0.30
Salt	0.39	0.30	0.30	0.30	0.28	0.26
DL-methionine	0.30	0.30	0.30	0.30	0.30	0.30
L-lysine	0.25	0.25	0.25	0.25	0.25	0.25
Sodium bicarbonate	0.10	0.10	0.10	0.10	0.10	0.10
L-threonine	0.07	0.07	0.07	0.07	0.07	0.07
Xylanase	0.01	0.01	0.01	0.01	0.01	0.01
Phytase	0.00	0.0075	0.0150	0.0225	0.0150	0.0225
Total	100.00	100.00	100.00	100.00	100.00	100.00
Calculated values						
Metabolizable energy (kcal/kg)3)	3,075	3,075	3,075	3,075	3,075	3,075
Crude protein (%)	19.82	19.82	19.82	19.82	19.82	19.82
Available phosphorus (%)	0.354	0.204	0.204	0.204	0.159	0.129
Calcium (%)	0.758	0.593	0.593	0.593	0.543	0.513
Sodium (%)	0.200	0.165	0.165	0.165	0.155	0.148
Chlorine (%)	0.284	0.232	0.232	0.232	0.217	0.206
Methionine+cystine (%)4)	0.826	0.826	0.826	0.826	0.826	0.826
Methionine (%)4)	0.684	0.684	0.684	0.684	0.684	0.684
Lysine (%)4)	1.131	1.131	1.131	1.131	1.131	1.131
Threonine (%)4)	0.735	0.735	0.735	0.735	0.735	0.735
Tryptophan (%)4)	0.214	0.214	0.214	0.214	0.214	0.214
Dietary electrolyte balance (mEq/kg)	199.72	199.31	199.31	199.31	199.20	199.12
Phytase (FTU/kg)5)	0	500	1,000	1,500	1,000	1,500
Xylanase (BXU/kg)6)	16,000	16,000	16,000	16,000	16,000	16,000
Analyzed values						
Phytic phosphorus (%)	0.210	0.270	0.260	0.240	0.250	0.270
Phytase (FTU/kg)	0	783	925	1,310	1,090	1,460
Xylanase (BXU/kg)	16,000	16,600	17,400	14,300	15,600	16,200

1) Matrix A (MT A): 0.165% Ca, 0.150% avP, and 0.035% Na; matrix B (MT B): 0.215% Ca, 0.195% avP, and 0.045% Na; matrix C (MT C): 0.245% Ca, 0.225% avP, and 0.053% Na.

2) Initial Vit+Min supplement–levels per kg of feed: 450.75 g methionine; 65.25 g choline; 2,750,000 UI vitamin A; 500,000 UI vitamin D_3_; 4,000 UI vitamin E; 375 mg vitamin K_3_; 300 mg vitamin B_1_; 1,125 mg vitamin B_2_; 500 mg vitamin B_6_; 4,000 mcg vitamin B_12_; 8,750 mg niacin; 2,300 mg pantothenic acid; 100 mg folic acid; 15 mg biotin; 7,500 mg iron; 2,250 mg cupper; 15 g manganese; 15 g zinc; 250 mg iodine; 62.5 mg selenium; 2,500 mg avilamycin; 10 g nicarbazin; 3,750 mg semduramicin. DEB: dietary electrolyte balance.

3) Nitrogen-corrected apparent metabolizable energy

4) Digestible amino acid.

5) Garantee levels per g of phytase: 5,000 FTU.

6) Garantee levels per g of xylanase: 16,000 BXU.

**Table 4:** Nutritional composition and calculated values of broiler experimental diets (34 to 42 days)

Items	Experimental diets1)					

	No phytase	Phytase 500 +MT A	Phytase 1,000 +MT A	Phytase 1,500 +MT A	Phytase 1,000 +MT B	Phytase 1,500 +MT C
Ingredients (%)						
Corn 7.88%	64.32	64.32	64.32	64.32	64.32	64.32
Soybean meal 46%	27.86	27.86	27.86	27.86	27.86	27.86
Soybean oil	3.36	3.36	3.36	3.36	3.36	3.36
Dicalcium phosphate	1.22	0.41	0.41	0.41	0.16	0.00
Kaolin	1.00	1.80	1.79	1.78	2.05	2.18
Limestone	0.83	0.92	0.92	0.92	0.94	0.97
Final Vit+Min supplement2)	0.30	0.30	0.30	0.30	0.30	0.30
Salt	0.37	0.29	0.29	0.29	0.26	0.25
DL-methionine	0.28	0.28	0.28	0.28	0.28	0.28
L-lysine	0.27	0.27	0.27	0.27	0.27	0.27
Sodium bicarbonate	0.10	0.10	0.10	0.10	0.10	0.10
L-threonine	0.08	0.08	0.08	008	0.08	0.08
Xylanase	0.01	0.01	0.01	0.01	0.01	0.01
Phytase	0.00	0.0075	0.0150	0.0225	0.0150	0.0225
Total	100.00	100.00	100.00	100.00	100.00	100.00
Calculated values						
Metabolizable energy (kcal/kg)3)	3,125	3,125	3,125	3,125	3,125	3,125
Crude protein (%)	18.40	18.40	18.40	18.40	18.40	18.40
Available phosphorus (%)	0.325	0.175	0.175	0.175	0.130	0.100
Calcium (%)	0.697	0.532	0.532	0.532	0.482	0.452
Sodium (%)	0.195	0.160	0.160	0.160	0.150	0.143
Chlorine (%)	0.277	0.224	0.224	0.224	0.210	0.199
Methionine+cystine (%)4)	0.774	0.774	0.774	0.774	0.774	0.774
Methionine (%)4)	0.649	0.649	0.649	0.649	0.649	0.649
Lysine (%)4)	1.060	1.060	1.060	1.060	1.060	1.060
Threonine (%)4)	0.689	0.689	0.689	0.689	0.689	0.689
Tryptophan (%)4)	0.194	0.194	0.194	0.194	0.194	0.194
Dietary electrolyte balance (mEq/kg)	184.78	184.38	184.38	184.38	184.26	184.18
Phytase (FTU/kg)5)	0	500	1,000	1,500	1,000	1,500
Xylanase (BXU/kg)6)	16,000	16,000	16,000	16,000	16,000	16,000
Analyzed values						
Phytic phosphorus (%)	0.240	0.260	0.250	0.240	0.230	0.250
Phytase (FTU/kg)	0	482	876	1,400	866	1,415
Xylanase (BXU/kg)	15,600	15,450	16,400	13,400	14,600	16,750

1) Matrix A (MT A): 0.165% Ca, 0.150% avP, and 0.035% Na; matrix B (MT B): 0.215% Ca, 0.195% avP, and 0.045% Na; matrix C (MT C): 0.245% Ca, 0.225% avP, and 0.053% Na.

2) Final Vit+Min supplement–levels per kg of feed: 1,104 mg pantothenic acid; 4.5 mg biotin; 3,000 mg cupper; 43.48 g choline; 10 g iron; 333.33 mg iodine; 20 g iodine; 20 g manganese; 301.95 g methionine; 1,500 mg niacin; 60 mg selenium; 900,000 UI vitamin A; 90 mg vitamin B_1_; 900 mcg vitamin B_12_; 300 mg vitamin B_2_; 120 mg vitamin B_6_; 150,000 UI vitamin D_3_; 1,500 UI vitamin E; 150 mg vitamin K_3_; 20 g zinc.

3) Nitrogen-corrected apparent metabolizable energy.

4) Digestible amino acid.

5) Garantee levels per g of Phytase: 5,000 FTU.

6) Garantee levels per g of Xylanase: 16,000 BXU.

**Table 5:** Performance of broilers fed diets supplemented with phytase from1to 21, 33, and 42 days of age

Variables	Experimental diets1)						p-value	SEM

	No phytase	Phytase 500 +MT A	Phytase 1,000 +MT A	Phytase 1,500 +MT A	Phytase 1,000 +MT B	Phytase 1,500 +MT C		
1–21 days								
FBW (g)	996	967	1,000	997	977	965	0.092	4.85
BWG (g/bird)	951	922	954	952	932	919	0.092	4.89
FI (g/bird)	1,338	1,299	1,324	1,322	1,338	1,314	0.697	7.64
FCR (g:g)	1.35	1.34	1.33	1.33	1.37	1.36	0.319	2.70
Liv (%)	98.8	98.3	99.4	98.3	98.3	97.7	0.780	0.33
1–33 days								
FBW (g)	2,105	2,096	2,128	2,135	2,083	2,117	0.375	7.75
BWG (g/bird)	2,060	2,050	2,083	2,090	2,038	2,071	0.375	7.78
FI (g/bird)	3,126	3,074	3,081	3,105	3,082	3,096	0.859	12.24
FCR (g:g)	1.48	1.47	1.45	1.45	1.48	1.46	0.081	0.01
Liv (%)	97.1	97.7	98.9	98.3	97.1	95.9	0.556	0.45
1–42 days								
FBW (g)	2,830	2,883	2,893	2,899	2,810	2,931	0.156	14.90
BWG (g/bird)	2.785	2.838	2.845	2.854	2.765	2.886	0.154	14.87
FI (g/bird)	4.657	4.687	4.666	4.685	4.600	4.735	0.619	20.75
FCR (g:g)	1.65	1.63	1.61	1.61	1.64	1.62	0.245	0.01
bwcFCR (g:g)	1.67	1.64	1.62	1.62	1.67	1.61	0.080	0.02
Liv (%)	93.7	96.6	94.9	94.2	95.4	93.7	0.816	0.66

SEM, standard error of the means; FBW, final body weight; BWG, body weight gain; FI, feed intake; FCR, feed conversion ratio; Liv, livability; bwcFCR, weight corrected feed conversion ratio.

1) Matrix A (MT A): 0.165% Ca; 0.150% avP; and 0.035% Na; matrix B (MT B): 0.215% Ca; 0.195% avP and 0.045% Na; matrix C (MT C): 0.245% Ca; 0.225% avP and 0.053% Na.

**Table 6:** Tibia ash and phosphorus content in defatted tibia of broilers at 21 days of age fed diets with different levels of phytase

Variables	Experimental diets1)						p-value	SEM

	No phytase	Phytase 500 +MT A	Phytase 1,000 +MT A	Phytase 1,500 +MT A	Phytase 1,000 +MT B	Phytase 1,500 +MT C		
Ash (%)	50.28	48.50	48.68	49.05	48.91	48.47	0.601	0.31
Tibia phosphorus (%)	8.50	8.04	8.40	8.18	8.15	8.26	0.730	0.08

SEM, standard error of the means.

1) Matrix A (MT A): 0.165% Ca, 0.150% avP, and 0.035% Na; matrix B (MT B): 0.215% Ca, 0.195% avP, and 0.045% Na; matrix C (MT C): 0.245% Ca, 0.225% avP, and 0.053% Na.

**Table 7:** Relative organ weights of broilers fed diets supplemented with phytase at 21 and 42 days of age

Variables2)	Experimental diets1)						p-value	SEM

	No phytase	Phytase 500 +MT A	Phytase 1,000 +MT A	Phytase 1,500 +MT A	Phytase 1,000 +MT B	Phytase 1,500 +MT C		
21 days of age								
Proventriculus (%)	0.58	0.56	0.57	0.58	0.60	0.58	0.955	0.01
Gizzard (%)	2.18	2.05	1.88	2.20	2.25	2.20	0.264	0.05
Small intestine (%)	3.35	3.23	3.08	2.97	3.31	3.26	0.785	0.08
Pancreas (%)	0.34	0.36	0.32	0.37	0.36	0.31	0.499	0.01
Heart (%)	0.61	0.64	0.63	0.69	0.67	0.68	0.751	0.02
Liver (%)	2.56	2.34	2.62	2.61	2.52	2.51	0.730	0.05
42 days of age								
Proventriculus (%)	0.29	0.30	0.25	0.24	0.26	0.27	0.151	0.01
Gizzard (%)	1.31	1.22	1.21	1.17	1.21	1.17	0.551	0.02
Small intestine (%)	1.56	2.09	2.00	1.81	2.24	2.04	0.152	0.04
Pancreas (%)	0.20	0.18	0.16	0.19	0.21	0.16	0.092	0.01
Heart (%)	0.44	0.49	0.46	0.41	0.50	0.48	0.426	0.01
Liver (%)	1.56^b^	1.83^ab^	1.73^ab^	1.61^ab^	2.02^a^	1.78^ab^	0.035	0.05

SEM, standard error of the means.

1) Matrix A (MT A): 0.165% Ca, 0.150% avP, and 0.035% Na; matrix B (MT B): 0.215% Ca, 0.195% avP, and 0.045% Na; matrix C (MT C): 0.245% Ca, 0.225% avP, and 0.053% Na.

2) %: Relative weights calculated in relation to bird live weight.

a,b Means followed by different letters in the same line are different by Tukey test (p<0.05).

**Table 8:** Carcass characteristics and cut yields of broilers fed diets supplemented with phytase at 42 days of age

Variables2)	Experimental diets1)						p-value	SEM

	No phytase	Phytase 500 +MT A	Phytase 1,000 +MT A	Phytase 1,500 +MT A	Phytase 1,000 +MT B	Phytase 1,500 +MT C		
Carcass (g)	2,175	2,143	2,251	2,260	2,137	2,232	0.366	21.48
Breast (g)	862.30	813.10	879.70	885.90	814.50	854.80	0.373	12.32
Thigh+drumstick (g)	606.50	620.60	640.10	631.60	623.10	632.40	0.706	6.12
Wings (g)	213.80	218.90	223.90	229.30	224.00	226.60	0.562	2.54
Head	135.44	153.00	156.22	156.78	168.22	140.87	0.444	2.70
Feet (g)	112.90	120.00	114.20	115.90	110.10	116.60	0.345	1.30
Back (g)	487.10	486.60	494.90	506.00	469.10	512.50	0.425	6.31
Abdominal fat (g)	38.60	43.90	39.50	43.10	42.50	42.00	0.944	1.69
Carcass (%)	74.63	73.74	79.06	75.40	75.32	76.99	0.262	0.68
Breast (%)	39.57	37.77	39.06	39.19	38.15	38.17	0.458	0.30
Thigh+drumstick (%)	27.92	29.06	28.46	27.99	29.16	28.37	0.413	0.21
Wings (%)	9.84	10.22	9.96	10.15	10.48	10.17	0.354	0.09
Head (%)	6.98	7.11	6.90	6.88	6.69	6.16	0.304	0.33
Feet (%)	5.19	5.62	5.09	5.13	5.15	5.24	0.068	0.06
Back (%)	22.43	22.79	21.97	22.34	21.92	23.01	0.628	0.21
Abdominal fat (%)	1.79	2.07	1.75	1.90	1.98	1.86	0.836	0.07

SEM, standard error of the means.

1) Matrix A (MT A): 0.165% Ca, 0.150% avP, and 0.035% Na; matrix B (MT B): 0.215% Ca, 0.195% avP, and 0.045% Na; matrix C (MT C): 0.245% Ca, 0.225% avP, and 0.053% Na.

2) %: Cut yields in relation to bird live weight.
